# Effects of background mutations and single nucleotide polymorphisms (SNPs) on the *Disc1* L100P behavioral phenotype associated with schizophrenia in mice

**DOI:** 10.1186/1744-9081-10-45

**Published:** 2014-12-08

**Authors:** Yosefu Arime, Ryutaro Fukumura, Ikuo Miura, Kazuyuki Mekada, Atsushi Yoshiki, Shigeharu Wakana, Yoichi Gondo, Kazufumi Akiyama

**Affiliations:** Department of Biological Psychiatry and Neuroscience, Dokkyo Medical University School of Medicine, 800 Kitakobayashi, Mibu-machi, Shimotsuga-gun, Tochigi, 321-0293 Japan; Mutagenesis and Genomics Team, Riken BioResource Center, 3-1-1, Koyadai, Tsukuba-shi, Ibaraki, 305-0074 Japan; Technology and Developmental Team for Mouse Phenotype Analysis, RIKEN BioResourse Center, 3-1-1, Koyadai, Tsukuba-shi, Ibaraki, 305-0074 Japan; Experimental Animal Division, RIKEN BioResourse Center, 3-1-1, Koyadai, Tsukuba-shi, Ibaraki, 305-0074 Japan

**Keywords:** Disrupted-in-schizophrenia 1 (DISC1), ENU mutagenesis, Locomotor activity, Methamphetamine, Social interaction, Whole-exome resequencing, Strain-specific SNPs

## Abstract

**Background:**

Disrupted-in-schizophrenia 1 (DISC1) is a promising candidate susceptibility gene for psychiatric disorders, including schizophrenia, bipolar disorder and major depression. Several previous studies reported that mice with *N*-ethyl-*N*-nitrosourea (ENU)-induced L100P mutation in *Disc1* showed some schizophrenia-related behavioral phenotypes. This line originally carried several thousands of ENU-induced point mutations in the C57BL/6 J strain and single nucleotide polymorphisms (SNPs) from the DBA/2 J inbred strain.

**Methods:**

To investigate the effect of *Disc1* L100P, background mutations and SNPs on phenotypic characterization, we performed behavioral analyses to better understand phenotypes of *Disc1* L100P mice and comprehensive genetic analyses using whole-exome resequencing and SNP panels to map ENU-induced mutations and strain-specific SNPs, respectively.

**Results:**

We found no differences in spontaneous or methamphetamine-induced locomotor activity, sociability or social novelty preference among *Disc1* L100P/L100P, L100P/+ mutants and wild-type littermates. Whole-exome resequencing of the original G1 mouse identified 117 ENU-induced variants, including *Disc1* L100P *per se*. Two females and three males from the congenic L100P strain after backcrossing to C57BL/6 J were deposited to RIKEN BioResource Center in 2008. We genotyped them with DBA/2 J × C57BL/6 J SNPs and found a number of the checked SNPs still remained.

**Conclusion:**

These results suggest that causal attribution of the discrepancy in behavioral phenotypes to the Disc1 L100P mutant mouse line existing among different research groups needs to be cautiously investigated in further study by taking into account the effect(s) of other ENU-induced mutations and/or SNPs from DBA/2 J.

**Electronic supplementary material:**

The online version of this article (doi:10.1186/1744-9081-10-45) contains supplementary material, which is available to authorized users.

## Background

Schizophrenia is a devastating mental disorder with a significant genetic component that affects approximately 0.5–1.0% of the general population. Based on genetic epidemiological studies, it was estimated that schizophrenia has a heritability of 60-80% [1, 2]. Disrupted-in-schizophrenia 1 (*DISC1*) is one of the candidate susceptibility genes for a spectrum of major psychiatric disorders. DISC1 was originally identified on chromosome 1 by analyzing a large Scottish pedigree showing a heavy burden of major psychiatric disorders associated with balanced chromosomal translocation (1:11)(q42.1:q14.3) [3, 4]. *DISC1* is implicated in the genetic risk for many aspects of quantitative cognitive traits of psychotic patients [5–8]. Accumulating evidence indicates that DISC1 acts as a scaffold protein that in concert with numerous interacting proteins regulates neurogenesis, neuronal migration, neurite outgrowth, and signal transduction [9–11].

Mouse models for the genetic mutation of *Disc1* are of relevance for unraveling the impact of gene disruption on neural integrity associated with schizophrenia. A number of conditional transgenic mouse models overexpressing truncated versions of human DISC1 protein under various exogenous promoters have been reported. In these models, truncated versions of human DISC1 protein are overexpressed either constitutively [12–14] or transiently during early postnatal life [15–18]. A mouse strain carrying a truncating lesion (25-bp deletion in exon 6) in the endogenous *Disc1* orthologue has also been reported [19–21]. Although these mouse models display a range of psychiatric disease-related behavioral phenotypes, many have been inconsistent across studies due to differences in behavior tests and experimental design and the specific genetic aberrations.

An alternative method for generation of a mouse *Disc1* model is to screen an archive of G1 mice carrying many *N*-ethyl-*N*-nitrosourea (ENU)-induced mutations. With this approach, it is feasible to identify an allelic series of ENU-induced mutations in a particular gene, in this case *Disc1*, as a gene-driven approach based on a priori assumptions of the function of a gene of interest [22, 23]. Genomic DNA libraries from thousands of G1 mice born to ENU-mutagenized C57BL/6 J male mice were screened for a point mutation in *Disc1*, one of which was subsequently identified as conferring a L100P amino acid substitution in exon 2 [24]. Roder and his collaborators reported that the *Disc1* L100P mouse line showed schizophrenia-like behavior, such as increased basal and amphetamine-induced open field activity, and deficits in prepulse inhibition (PPI) [24, 25]. However, other independent research groups observed neither an increase in basal locomotor activity nor abnormal PPI [26, 27].

Because ENU generates multitude, randomly distributed, single-base point mutations throughout the entire mouse genome, ENU mutant mouse lines may harbor unknown functional mutations other than those are assumed to contribute to the phenovariance of interest [22, 23, 28, 29]. Additionally, ENU-mutagenized C57BL/6 J male mice at RIKEN were out-crossed to untreated DBA/2 J female mice to produce the original progeny (G1). Although heterozygous mice resulting from repeated backcrossing to the C57BL/6 J background were finally intercrossed to generate L100P/L100P homozygous mice, remaining ENU-induced mutations outside *Disc1* and/or single nucleotide polymorphisms (SNPs) from DBA/2 J background may confound the causal relationship between L100P mutation and the behavioral phenotype. Disclosure of this information would provide a justification for the usage of ENU-mutagenesis to fully explain the phenotypic manifestation of the main causative mutation. Herein, we performed three experiments to better understand behavioral phenotypes of *Disc1* L100P mice with reference to our new genetic data. First, we investigated the genetic background of the L100P strain that was reversely-deposited back to the Experimental Animal Division (EAD), RIKEN BioResource Center (BRC) by Roder’s group [24]. The genetic background of the deposited L100P/L100P homozygous pairs and their offspring born via intercrossing between the homozygotes were investigated. Second, L100P/L100P, L100P/+ and wild-type littermates (+/+), which were derived from the deposited L100P/L100P homozygous mice mated with the C57BL/6 J mice (details in Materials and Methods), were examined for schizophrenia-related behavioral phenotypes using open field and social interaction tests. Third, we conducted whole-exome resequencing analysis of the original G1 genome to screen for ENU-induced mutations in addition to the *Disc1* L100P mutation. The results obtained were discussed in light of the validity and issue of *Disc1* L100P mutant model.

## Methods

### Animals

Male and female homozygous *Disc1* L100P/L100P mutant mice backcrossed to C57BL/6 J were deposited at the EAD at RIKEN BRC (Tsukuba, Japan, http://www.brc.riken.jp/lab/animal/en/) by another research group [24], and their offspring was bred to maintain the homozygosity (L100P/L100P). These L100P/L100P mutant male mice (Disc1 < Rgsc1390>) were obtained from RIKEN and then backcrossed to an inbred C57BL/6 J female (Japan Clea Co., Tokyo Japan) for one generation. The resultant L100P/+ progeny were intercrossed to generate L100P/L100P, L100P/+ and +/+ littermates. Mice were weaned at postnatal day 25–28 and segregated by sex; were housed 2–4 per cage in a temperature-controlled (25 ± 1°C) and light-controlled room (light on 0600–1800 h) in plastic cages with *ad libitum* access to food and water. PCR-based genotyping was conducted with a primer pair (common forward primer: 5'-CCTGTCCCAAGGACTGGCATC-3'; reverse primer for L100L wild-type: 5'-CAGGGACAAGGGAGCTCTTCA-3'; reverse primer for L100P mutants: 5'- CAGGGACAAGGGAGCTCTTCG-3') and genomic DNA extracted from tail biopsies using Hot Sodium Hydroxide and Tris (HotSHOT) method [30]. Age-matched male L100P/L100P, L100P/+ and +/+ mice (12–16 weeks old) were compared in behavioral analyses. To compare wild-type littermates with inbred C57BL/6 J mice, 12–16-week-old male C57BL/6 J mice were purchased from Japan Clea Co. (Tokyo, Japan). All experiments were performed in accordance with the Guidelines for Care and Use of Laboratory Animals, Dokkyo University School of Medicine and conformed to all Japanese federal rules and guidelines.

### Drug treatment

Methamphetamine hydrochloride (Dainippon Pharmaceutical Co., Osaka, Japan) was dissolved in saline and administered subcutaneously (s.c.) in a volume of 10 ml/kg.

### Behavioral analysis

All behavioral analyses were recorded with a CCD camera and analyzed using video tracking software (ANY-maze ver. 4.82; Stoelting Co., USA). The apparatus was illuminated at approximately 300 lux. Mice were transferred to the experimental site at least 30 min before testing.

### Spontaneous locomotor activity

Mice were placed individually in grey plastic cages (42 × 42 × 30 cm). Spontaneous locomotor activity was recorded in 5-min sessions during a 90-min test period, with distance traveled as the primary outcome measure.

### Methamphetamine-induced locomotor activity

Mice were placed individually in grey plastic cages (42 × 42 × 30 cm) for a 30-min habituation session and then injected subcutaneously with methamphetamine (0.2, 0.5 or 1.0 mg/kg) or saline. Locomotor activity was recorded continuously during the 30-min habituation period and for 60 min after injection of saline or methamphetamine. To verify effects of genetic characteristics on behavioral phenotypes, methamphetamine-induced locomotor activity was compared among mice in the following experiments. In experiment 1, effects of L100P on methamphetamine-induced locomotor activity were compared in age-matched male littermates (L100P/L100P, L100P/+ and +/+). In experiment 2, effects of genetic background on methamphetamine-induced locomotor activity were tested by comparing inbred C57BL/6 J mice with the results of wild-type littermate born to *Disc1* L100P mutant line obtained from the experiment 1.

### Sociability and social novelty preference tests

Effects of the L100P point mutation on social behavior were assessed according to previously described methods [24, 31] with minor modifications. A three-chamber apparatus was constructed of clear acrylic sheets; each chamber had a size of 19 × 40 × 25 cm (width × depth × height). Each side chamber contained a wire-bar cup (Galaxy Cup, Spectrum Diversified Designs, Inc., OH, USA) placed on either side of the arena. Two dividing walls containing doors allowed access to each of the side chambers from the center chamber. The behavioral test consisted of three sessions: (1) habituation, (2) sociability and (3) social novelty preference. In the habituation session (1), the subject mouse was placed in the center chamber and allowed to freely explore all three chambers for 10 min. In the sociability session (2), following the habituation session, the subject mouse was introduced in the center chamber for 1 min with access to the side chambers blocked by white partitions. An unfamiliar male C57BL/6 J mouse (Stranger 1) was enclosed in the wire-bar cup in one of the side chambers. The location of Stranger 1 alternated among subject mice. On removal of partitions, the subject mouse was allowed to freely explore the entire apparatus for 10 min. Time spent within the interaction zone (an oblong area of 19 × 15 cm containing the wire-bar cup) and the number of entries into each chamber was measured. In the social novelty preference test (3) conducted after the sociability test, the subject mouse was placed in the center chamber for 1 min. An unfamiliar intruder male C57BL/6 J mouse (Stranger 2) was enclosed in the wire-bar cup in the other side chamber. Time spent in the interaction zones and the number of entries into each chamber was measured.

### SNP-typing for genetic background of L100P/L100P mutant mice

We genotyped 117 SNPs, which were openly available in the SNP database of RIKEN BRC (http://ja.brc.riken.jp/lab/jmc/mapping.html), distributed at approximately 15-cM intervals over the entire mouse genome in 8 mice; 3 males and 1 female of the L100P/L100P deposited mice to the RIKEN EAD and 1 male and 3 female progeny from the deposited pairs to distinguish between C57BL/6 J and DBA/2 J genetic backgrounds.

### Exome re-sequencing of the original G1 carrying the L100P mutation

We used the SureSelect Mouse All Exon Kit (Agilent, Santa Clara, CA, USA) to enrich whole exons from genomic DNA of the G1 mouse. We performed resequencing using SOLiD4 (Life Technologies, Carlsbad, CA, USA) as reported previously [32].

### Statistical analysis

Statistical analysis was conducted using SPSS software (ver. 19, IBM Japan). Data were analyzed using paired t-tests, one-way ANOVA, two-way ANOVA or two-way repeated measures ANOVA as appropriate. The Greenhouse–Geisser correction for repeated measures was applied as necessary.

## Results

### Spontaneous locomotor activity

**Figure 1:**
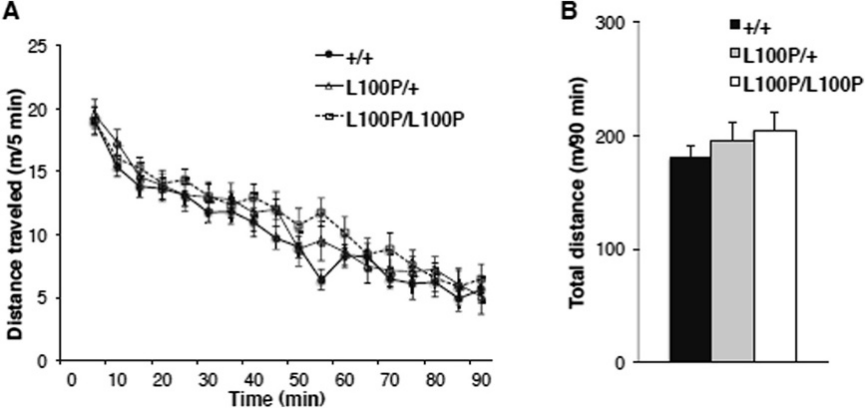
**Comparison of spontaneous locomotor activity in the open field. (A)** Distance traveled in 5 min bins during spontaneous locomotion by *Disc1* L100P/L100P mutants (n = 20), L100P/+ mutants (n = 18), and wild-type (+/+) littermates (n = 20). Repeated measures ANOVA revealed no significant genotype × time interaction. Data are expressed as mean ± SEM. **(B)** Total distance traveled over 90 min. ANOVA revealed no significant main effect of genotype. Data are expressed as mean ± SEM.

Spontaneous locomotor activity in the open field declined to a similar extent in all three genotypes (L100P/L100P, L100P/+ and +/+) over 90 min (Figure 1A). Two-way repeated measures ANOVA revealed no significant genotype × time interaction (F(20.169, 554.636) = 0.884, *p* = 0.609). Moreover, total distance traveled was similar among genotypes (Figure 1B). ANOVA revealed no main effect of genotype on total distance traveled (F(2, 55) = 0.785, *p* = 0.461).

### Methamphetamine-induced locomotor activity

**Figure 2:**
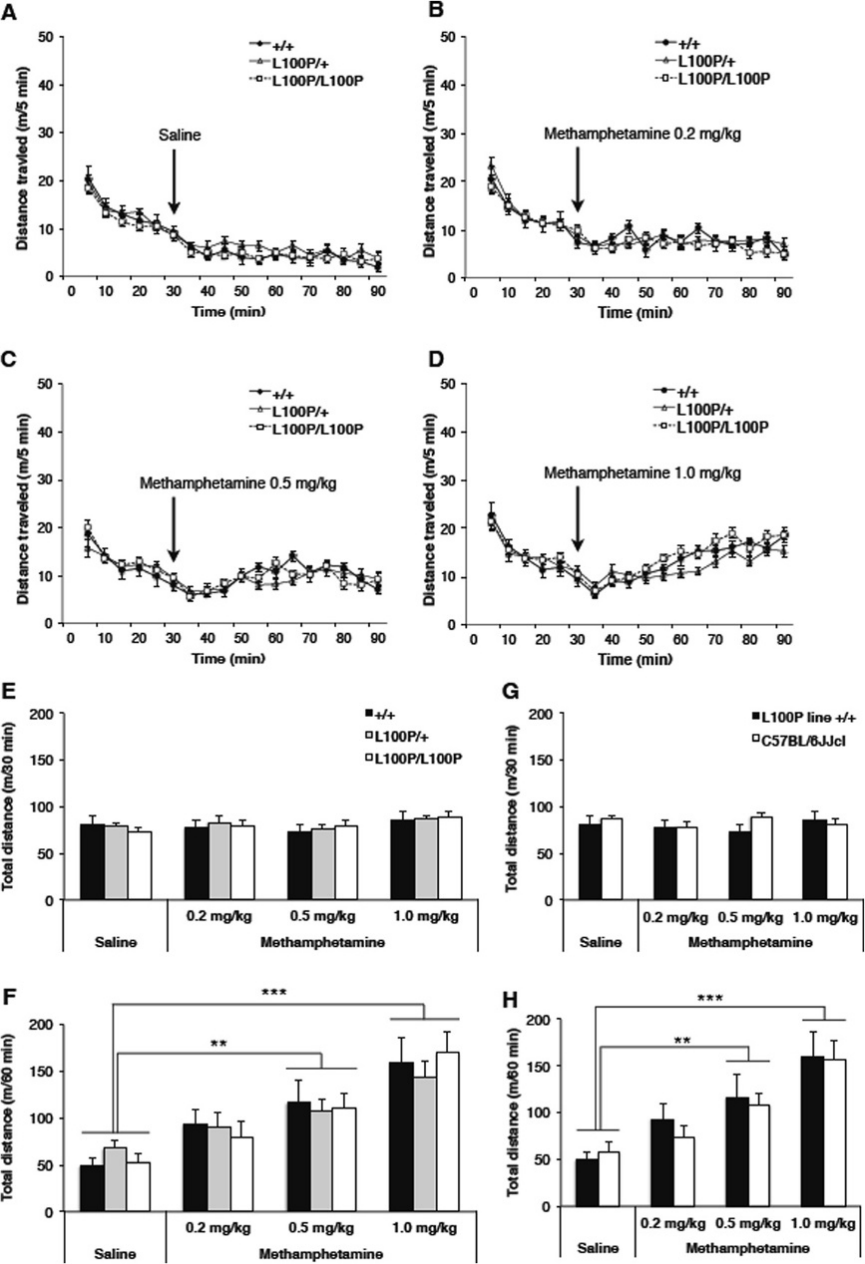
**Effect of methamphetamine on locomotor activity in the open field. (A–D)** Distance traveled in 5 min bins following methamphetamine or saline treatment. Repeated measures ANOVA revealed no significant genotype × time interactions among *Disc1* L100P/L100P (n = 11–12), L100P/+ (n = 11–13), and +/+ littermates (n = 8–9). Data are expressed as mean ± SEM. **(E)** Total distance traveled during 30 min before drug injection. ANOVA revealed no significant genotype × drug interaction or a main effect of genotype. **(F)** Total distance traveled during 60 min after drug injection. ANOVA revealed a significant main effect of the drug. Methamphetamine elicited hyperlocomotion in a dose-dependent manner in all three genotypes. **(G, H)** Effect of methamphetamine on the locomotor activity of inbred C57BL/6 J mice and +/+ littermates derived from *Disc1* L100P mutant line in the open field. **(G)** Total distance traveled for 30 min before drug injection. ANOVA revealed no significant genetic background × drug interaction or a main effect of genetic background. **(H)** Total distance traveled over 60 min after drug injection. ANOVA revealed a significant main effect of drug. Methamphetamine elicited hyperlocomotion in a dose-dependent manner in both two groups. L100P line +/+ in **(G, H)** and +/+ in **(E, F)** were the same mice for each treatment group. Data are expressed as mean ± SEM. **p < 0.01 or ***p < 0.001 compared with the saline-treated group.

There was no difference among genotypes in locomotor activity during either the habituation period or post-injection period (Figure 2A–D). ANOVA indicated no significant genotype × drug interaction on total distance traveled (F(6, 117) = 0.214, *p* = 0.972), no main effect of drug treatment (F(3, 117 = 1.824, *p* = 0.147) and no main effect of genotype (F(2, 117) = 0.098, *p* = 0.907) during 30-min habituation (Figure 2E). Similarly, there was no significant genotype × drug interaction on total distance traveled among groups (F(6, 117) = 0.433, *p* = 0.856) or a main effect of genotype (F(2, 117) = 0.015, *p* = 0.985) during 60 min after drug injection (Figure 2F). Methamphetamine (0.5 and 1.0 mg/kg) significantly increased total distance traveled in all genotypes. ANOVA revealed a significant effect of drug treatment (F(3, 117) = 19.844, *p* < 0.001); Tukey *post hoc* analysis showed a significant effect of 0.5 mg/kg (*p* < 0.01) and 1.0 mg/kg (*p* < 0.001) methamphetamine versus saline.

To test for effects of putatively existing genetic elements inherited from the DBA/2 J background on spontaneous and methamphetamine-induced locomotion, we compared wild-type progeny born to *Disc1* L100P/+ mutants and inbred C57BL/6 J mice. There was no difference in either spontaneous or methamphetamine-induced locomotor activity (Figure 2G–H). ANOVA revealed no significant drug × genetic background interaction in either the 30-min habituation period (F(3,59) = 0.779, *p* = 0.511) or the 60-min post-injection period (F(3, 59) = 0.198, *p* = 0.898). There was no main effect of genetic background on locomotor activity in either the habituation period (F(1,59) = 0.806, *p* = 0.373) or the post-injection period (F(1,59) = 0.195, *p* = 0.660). There was no main effect of drug treatment on total distance traveled during the habituation period (F(3,59) = 0.244, *p* = 0.866) (Figure 2G). Methamphetamine (0.5 and 1.0 mg/kg) significantly increased locomotor activity in both wild-type progeny of *Disc1* L100P/+ mice and inbred C57BL/6 J mice (Figure 2H). There was a significant main effect of drug treatment on total distance traveled after the drug administration (F(3, 59) = 12.114, p < 0.001) and significant effects of both methamphetamine 0.5 mg/kg (p < 0.01) and 1.0 mg/kg (p < 0.001) versus saline as revealed by Dunnett’s T3 *post hoc* analysis.

### Sociability and social preference test

**Figure 3:**
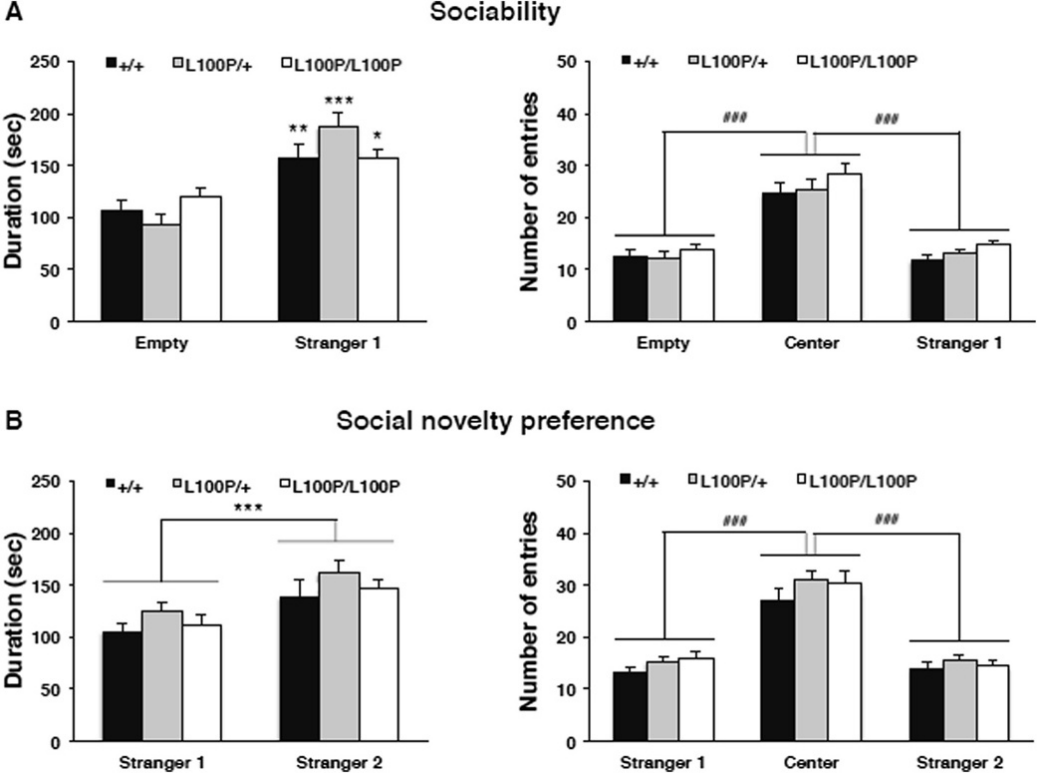
**Comparison of sociability and social novelty preference among** ***Disc1*** **L100P/L100P, L100P/+, and +/+ littermates. (A)** Sociability: mean time spent near the empty cage (Empty) or the cage containing the unfamiliar mouse (Stranger 1) (left), and the number of entries into each chamber (right). **(B)** Mean time spent near the cage containing the first unfamiliar mouse (Stranger 1) or a new unfamiliar mouse (Stranger 2) (left), and the number of entries into each chamber (right). All values are expressed as mean ± SEM for each group. ^*^ *p* < 0.05, ^**^ *p* < 0.01 or ^***^ *p* < 0.001 versus time spent near the unfamiliar mouse (Stranger 1). ^###^ *p* < 0.001 versus the number of entries into the center chamber.

In the sociability session, two-way ANOVA revealed a significant interaction between genotype and the chambers in time spent in the interaction zones (F(2, 50 = 3.495, *p* < 0.05). *Post hoc* analyses revealed no significant main effect of genotype for either side, but did show a significant main effect of preference for the stranger mouse (Stranger 1) for all genotypes (+/+: *p* < 0.01, L100P/+: *p* < 0.001, L100P/L100P: *p* < 0.05) on stay duration (Figure 3A). Two-way repeated measures ANOVA showed neither a significant genotype × chamber interaction for the number of entries nor a main effect of genotype, but did show a significant main effect of the chamber (F(1.533, 76.638) = 403.012, *p* < 0.001). *Post hoc* analyses revealed a significant main effect of preference for the center zone for the combined group (Empty: *p* < 0.001, Stranger 1: *p* < 0.001), but the number of transitions into the chamber containing Stranger 1 did not differ from the number of transitions into the empty chamber. In the social novelty preference session, two-way repeated measures ANOVA showed neither a significant genotype × side interaction in time spent in the interaction zones nor a significant main effect of genotype, but did reveal a significant main effect of both sides (F(1, 50) = 17.247, *p* < 0.001). *Post hoc* analyses revealed a significant main effect of preference for the unfamiliar intruder (Stranger 2) compared with the by-then familiar mouse (Stranger 1) for the combined group (*p* < 0.001) (Figure 3B). Two-way repeated measures ANOVA showed neither a significant genotype × chamber interaction for the number of entries nor a significant main effect of genotype, but did show a significant main effect of the chamber (F(1.669, 83.456) = 346.789, *p* < 0.001). *Post hoc* analyses revealed a significant main effect of preference for the center zone for the combined group (Stranger 1: *p* < 0.001, Stranger 2: *p* < 0.001), but the number of transitions into the chamber containing Stranger 1 did not differ from the number of transitions into the chamber containing Stranger 2.

### SNP typing for genetic background of L100P/L100P mutant mouse line (Disc1 < Rgsc1390>)

Despite backcrosses, all the genotyped eight L100P/L100P homozygotes harbored DBA/2 J-derived SNP alleles, which were either homozygous or heterozygous variant (Additional file 1: Figure S1). Genome-wide SNP panel analysis revealed that the numbers of SNPs indicative of homozygosity or heterozygosity for DBA/2 J ancestry varied among individual mice. Each mouse harbored 1–5 homozygous and 8–12 heterozygous loci for DBA/2 J-derived SNP alleles (Additional file 2: Table S1).

### Exome resequencing of the original G1 genome carrying L100P mutation

There was no single nucleotide mutation in the *Disc1* exons other than L100P. In addition to the L100P mutation, exome resequencing analysis revealed 117 single nucleotide mutations in the exome and exome-flanking regions of the genomic DNA from the original G1 mouse, the F1 progenitor born to an ENU-treated G0 mouse (Table 1). *Disc1* L100P and two other mutations (in *Rab11fip1*, known as Rab-coupling protein and *Nr3c2*) were found in chromosome 8. Other variants included synonymous coding variants (5), nonsynonymous coding (27 including *ryr3*, encoding the intracellular Ca2+ release channel ryanodine receptor 3 and *Rab11fip1*), knockout-equivalent nonsense (*Als2* and *Ttll1*), essential splice site variants (3), 5′ UTR (3, including *Dyx1c1*, a susceptibility gene for dyslexia), 3′ UTR (9), and intronic mutations (49). Base substitution spectra of the 117 ENU-induced mutations were A/T to T/A transversions (26.5%), A/T to G/C transitions (39.3%), G/C to A/T transitions (18.8%), A/T to C/G transversions (6.8%), and G/C to T/A transversions (8.5%).

**Table 1:** **List of ENU-induced mutations in G1 mouse of Disc1<Rgsc1390>**

Gene symbol	Entrez Gene ID	Chromosome	Position (MM9)	Base change	Spectrum	Classification	Amino acid substitution
***Pkhd1***	241035	1	20,530,613	T → A	AT to TA transversions	Intronic	
***Trak2***	70827	1	58,975,614	A → G	AT to GC transitions	Essential splice site	
***Als2***	74018	1	59,235,933	A → T	AT to TA transversions	Stop gained	Y- > X
***Ngef***	53972	1	89,379,202	A → T	AT to TA transversions	Nonsysnonymous	I- > N
***2310035C23Rik***	227446	1	107,592,127	A → G	AT to GC transitions	Intronic	
***Ikbke***	56489	1	133,172,385	C → A	GC to TA transversions	Nonsysnonymous	A- > S
***Cdc73***	214498	1	145,542,523	A → T	AT to TA transversions	Nonsysnonymous	S- > T
***Nmnat2***	226518	1	154,936,083	A → T	AT to TA transversions	Intronic	
***Atf6***	226641	1	172,724,783	G → A	GC to AT transitions	Intronic	
***Gm5706***	*435657*	1	184,322,753	A → G	AT to GC transitions	Genic	
***Iars2***	381314	1	187,127,260	A → T	AT to TA transversions	Nonsysnonymous	I- > N
***Dhtkd1***	209692	2	5,831,429	A → G	AT to GC transitions	Synonymous	
***Cacna1b***	12287	2	24,506,396	A → G	AT to GC transitions	Intronic	
***Dolk***	227697	2	30,140,520	A → G	AT to GC transitions	Synonymous	
***Lrp1b***	94217	2	40,854,333	A → C	AT to CG transversions	Intronic	
***Neb***	17996	2	52,114,402	C → A	GC to TA transversions	Nonsysnonymous	R- > L
***Gm13559***	*674940*	2	58,593,845	A → G	AT to GC transitions	Genic	
***Ttn***	22138	2	76,553,805	C → T	GC to AT transitions	Nonsysnonymous	M- > I
***Olfr1270***	258987	2	89,989,253	T → C	AT to GC transitions	Synonymous	
***Ext2***	14043	2	93,558,064	C → T	GC to AT transitions	Intronic	
***Ryr3***	20192	2	112,701,983	C → A	GC to TA transversions	Nonsysnonymous	G- > W
***Fmn1***	14260	2	113,528,228	T → A	AT to TA transversions	Intronic	
***Sel1l2***	228684	2	140,071,126	C → T	GC to AT transitions	Nonsysnonymous	A- > T
***Setd7***	73251	3	51,334,212	T → C	AT to GC transitions	Intronic	
***Ift80***	68259	3	68,794,587	A → T	AT to TA transversions	Intronic	
***Asb17***	66772	3	153,516,522	G → A	GC to AT transitions	3' UTR	
		4	8,394,708	T → C	AT to GC transitions	Intergenic	
***Esrp1***	207920	4	11,277,315	G → T	GC to TA transversions	Intronic	
***Gm136***	214568	4	34,699,557	C → T	GC to AT transitions	Nonsysnonymous	A- > T
***Nfx1***	74164	4	40,952,108	T → A	AT to TA transversions	Intronic	
***Gm12608***	*664785*	4	89,109,036	T → A	AT to TA transversions	Genic	
***Tek***	21687	4	94,516,374	T → A	AT to TA transversions	Nonsysnonymous	S- > T
***Dnajc6***	72685	4	101,279,103	C → T	GC to AT transitions	Intronic	
***Txndc12***	66073	4	108,533,802	T → A	AT to TA transversions	Intronic	
***Gm12901***	*194197*	4	122,935,965	T → C	AT to GC transitions	Genic	
***Tmprss11e***	243084	5	87,156,236	C → T	GC to AT transitions	Intronic	
***Ccng2***	12452	5	93,697,763	T → C	AT to GC transitions	Nonsysnonymous	I- > T
***Calcr***	12311	6	3,650,125	A → G	AT to GC transitions	Essential splice site	
***C130060K24Rik***	243407	6	65,406,380	A → G	AT to GC transitions	Nonsysnonymous	K- > E
***Wnk1***	232341	6	119,956,300	A → T	AT to TA transversions	Intronic	
***Vwf***	*22371*	6	125,576,322	G → A	GC to AT transitions	Nonsysnonymous	M- > I
***Klrk1***	27007	6	129,566,794	G → A	GC to AT transitions	Intronic	
***Olfr1336***	258917	7	6,413,462	A → T	AT to TA transversions	Nonsysnonymous	T- > S
***Nlrp9a***	233001	7	27,358,923	A → C	AT to CG transversions	Nonsysnonymous	E- > A
***Arhgap33***	233071	7	31,315,480	C → T	GC to AT transitions	Synonymous	
***Gucy2d***	14918	7	105,600,256	A → T	AT to TA transversions	Exonic	
		7	146,105,909	T → C	AT to GC transitions	Intergenic	
***Rab11fip1***	75767	8	28,263,563	G → A	GC to AT transitions	Nonsysnonymous	P- > L
***Nr3c2***	110784	8	79,741,251	T → C	AT to GC transitions	Intronic	
***Disc1***	244667	8	127,611,597	T → C	AT to GC transitions	Nonsysnonymous	L- > P
***Prdm10***	382066	9	31,142,994	T → C	AT to GC transitions	Intronic	
***Olfr909***	100043200	9	38,331,305	T → C	AT to GC transitions	Upstream	
***Olfr44***	258716	9	39,291,849	T → G	AT to CG transversions	3' UTR	
***Pou2f3***	18988	9	42,984,368	C → T	GC to AT transitions	Essential splice site	
***Dyx1c1***	67685	9	72,806,667	G → A	GC to AT transitions	5' UTR	
***Unc13c***	208898	9	73,582,186	T → C	AT to GC transitions	Intronic	
***Senp6***	215351	9	79,969,034	T → G	AT to CG transversions	Intronic	
***Cep63***	28135	9	102,521,076	T → C	AT to GC transitions	Intronic	
***Heca***	380629	10	17,635,364	T → C	AT to GC transitions	Nonsysnonymous	N- > S
***Olig3***	94222	10	19,077,712	A → G	AT to GC transitions	3' UTR	
***Sobp***	109205	10	42,742,835	A → T	AT to TA transversions	Intronic	
***Serinc1***	56442	10	57,245,307	A → G	AT to GC transitions	Intronic	
***Sf3a2***	20222	10	80,263,982	A → G	AT to GC transitions	Nonsysnonymous	D- > G
***Cyp27b1***	13115	10	126,485,434	T → C	AT to GC transitions	Nonsysnonymous	V- > A
***Tns3***	319939	11	8,449,105	T → A	AT to TA transversions	5' UTR	
***Il5***	16191	11	53,537,612	C → T	GC to AT transitions	3' UTR	
***4930404A10Rik***	74847	11	54,185,249	T → A	AT to TA transversions	Nonsysnonymous	S- > R
***Ulk2***	29869	11	61,615,130	A → T	AT to TA transversions	Intronic	
***Slc13a5***	237831	11	72,061,131	C → T	GC to AT transitions	Intronic	
***Ap2b1***	71770	11	83,183,124	G → A	GC to AT transitions	Intronic	
***Lrrc46***	69297	11	96,902,597	T → A	AT to TA transversions	5' UTR	
***Erbb2***	13866	11	98,297,347	T → C	AT to GC transitions	Synonymous	
***Krt40***	406221	11	99,401,666	A → G	AT to GC transitions	Intronic	
***Syngr2***	20973	11	117,674,256	T → A	AT to TA transversions	Intronic	
***Gm9229***	*668539*	12	18,193,251	C → A	GC to TA transversions	Genic	
***Mark3***	17169	12	112,885,660	T → C	AT to GC transitions	Intronic	
***Igh***	*111507*	12	115,853,922	T → A	AT to TA transversions	Genic	
***Tcrg-V4***	*21638*	13	19,282,580	A → C	AT to CG transversions	Genic	
***Aoah***	27052	13	21,003,081	A → T	AT to TA transversions	Intronic	
***Slc6a3***	13162	13	73,682,459	T → C	AT to GC transitions	Intronic	
***Gtf2h2***	23894	13	101,240,804	T → A	AT to TA transversions	Intronic	
***Ccdc125***	76041	13	101,449,272	A → G	AT to GC transitions	Nonsysnonymous	D- > G
***3425401B19Rik***	100504518	14	33,476,075	A → G	AT to GC transitions	Nonsysnonymous	I- > T
***Mcpt1***	17224	14	56,637,381	G → T	GC to TA transversions	Intronic	
***Atp8a2***	50769	14	60,266,756	A → G	AT to GC transitions	Nonsysnonymous	Y- > H
***Sorbs3***	20410	14	70,586,596	A → T	AT to TA transversions	Intronic	
***Epsti1***	108670	14	78,397,889	G → T	GC to TA transversions	Intronic	
***Diap3***	56419	14	87,384,824	G → T	GC to TA transversions	Intronic	
***Vps13b***	666173	15	35,570,473	A → G	AT to GC transitions	Intronic	
***Ubr5***	70790	15	37,898,017	A → G	AT to GC transitions	3' UTR	
***Micall1***	27008	15	78,960,399	G → A	GC to AT transitions	Intronic	
***Cyp2d12***	380997	15	82,387,610	A → T	AT to TA transversions	Intronic	
***Ttll1***	319953	15	83,320,020	G → A	GC to AT transitions	Stop gained	R- > X
***Prkag1***	19082	15	98,643,692	A → G	AT to GC transitions	3' UTR	
***Gm7638***	*665450*	16	11,185,895	C → T	GC to AT transitions	Genic	
***Myh11***	17880	16	14,269,121	A → G	AT to GC transitions	Intronic	
***Gm6931***	*628903*	16	49,425,860	T → A	AT to TA transversions	Genic	
		16	80,727,008	A → C	AT to CG transversions	Intergenic	
***Olfr125***	258287	17	37,972,895	T → C	AT to GC transitions	Nonsysnonymous	I- > T
***Enpp5***	83965	17	44,222,094	T → C	AT to GC transitions	Intronic	
***Gm7334***	*654432*	17	50,838,499	T → C	AT to GC transitions	Genic	
***Sult1c1***	20888	17	54,101,361	T → C	AT to GC transitions	3' UTR	
***2700099C18Rik***	77022	17	95,163,195	C → T	GC to AT transitions	Genic	
***Fhod3***	225288	18	25,248,741	A → G	AT to GC transitions	Nonsysnonymous	N- > S
***Kdm3b***	277250	18	34,982,966	T → G	AT to CG transversions	Intronic	
***Pcdhb6***	93877	18	37,493,679	A → G	AT to GC transitions	Upstream	
***Pcdhga1***	*93709*	18	37,912,242	C → T	GC to AT transitions	Intronic	
***Spink10***	328971	18	62,820,759	G → T	GC to TA transversions	3' UTR	
***Cst6***	73720	19	5,344,061	A → T	AT to TA transversions	Intronic	
***Ganab***	14376	19	8,987,570	T → A	AT to TA transversions	Intronic	
***Cpsf7***	269061	19	10,607,448	G → T	GC to TA transversions	Intronic	
***Ms4a5***	269063	19	11,358,379	A → T	AT to TA transversions	Upstream	
***Ms4a14***	*383435*	19	11,382,160	A → T	AT to TA transversions	Genic	
***Tjp2***	21873	19	24,194,331	T → C	AT to GC transitions	Intronic	
***Cyp2c29***	13095	19	39,405,010	T → C	AT to GC transitions	3' UTR	
***Tll2***	24087	19	41,257,593	T → C	AT to GC transitions	Intronic	
***Sorcs3***	66673	19	48,768,473	A → C	AT to CG transversions	Nonsysnonymous	I- > L

## Discussion

The present study compared schizophrenia-related behavior among L100P mutant mice (L100P/L100P and L100P/+) and their wild-type littermates, and inbred C57BL/6 J mice by testing spontaneous locomotor activity, methamphetamine-induced locomotor activity in the open field [33], and sociability/social novelty preference in the social interaction [34]. All of behavior was comparable among L100P/L100P, L100P/+ and wild-type littermates; the results were partially inconsistent with previous studies using mice originated from the same G1 founder [24, 25]. To assess effects of genetic background (C57BL/6 J vs DBA/2 J), we conducted comprehensive genotyping of the original G1 mouse and its homozygous offspring obtained by backcrossing to C57BL/6 J, the same strain used for backcrossing in the previous report characterizing the L100P mutant [24]. Our genetic analyses demonstrated that both the L100P/L100P homozygous mice deposited by Roder’s group [24] into the EAD at RIKEN and their homozygous progeny still harbored a small number of SNPs inherited from the DBA/2 J strain (the mother of the G1 founder). Whole-exome resequencing of the original G1 genome also revealed an additional 116 single nucleotide variants induced by ENU.

*Disc1* L100P was originally identified in one G1 progeny of several thousand screened for *Disc1* mutants from a G1 male genomic DNA archive produced by breeding ENU-mutagenized C57BL/6 J males and untreated DBA/2JJcl females [24]. Live heterozygous mice carrying the L100P mutation were recovered by *in vitro* fertilization of C57BL/6 J eggs with the cryopreserved sperm of the corresponding G1 progeny. In the study reported by Clapcote et al. [24], mutant mice were then backcrossed to the C57BL/6 J background for at least six generations before intercrossing L100P/+ mice to generate homozygous mutants for behavioral tests. In theory, the backcrossed congenic mutant strain should have lost most of the other ENU-induced mutations and SNPs derived from DBA/2 J; however, the rate of SNP and mutant loss per generation has not been examined experimentally.

DBA/2 J and C57BL/6 J inbred strains were shown to exhibit differential sensitivity to psychostimulants [35, 36] without significant differences in basal locomotor activity or sociability [31]. Thus, residual DBA/2 J SNPs, if any still exist in the analyzed mice, could conceivably influence the behavioral test results. Genomic analysis revealed a significant number of DBA/2 J SNPs remaining in the backcrossed *Disc1* L100P mice. Among 117 loci tested, nineteen were still polymorphic in the eight L100P/L100P mice deposited at RIKEN and their progeny (Additional file 2: Table S1). The average frequency of DBA/2 J SNP alleles in the eight L100P/L100P mice was 6.63% (5.56%–8.12%, Additional file 2: Table S1); a rate of genetic vestige was 4.25-fold higher than the theoretical estimate of 1.56% [24]. The question arises as to whether any single DBA/2 J SNP or combination influences *Disc1* L100P locomotor activity in the open field compared with the congenic background strain. An important feature of the present study was the inclusion of commercially available inbred C57BL/6 J mice in addition to wild-type littermates derived from *Disc1* L100P mutants to assess effects of genetic background on behavioral phenotype. This experimental design could enhance the sensitivity for detecting effects of genetic background and ENU-induced mutations. However, our behavioral analyses showed that neither basal nor methamphetamine-induced locomotor activity differed between wild-type mice derived from *Disc1* L100P mutants and inbred C57BL/6 J mice. This finding suggests that locomotor activity is not measurably affected by residual DBA/2 J SNPs. Subsequently, using the exome resequencing analysis, we identified 116 previously unreported mutations in the exome of the original G1 male, although L100P was the only single mutation found in the coding sequence of the *Disc1* gene. It has been estimated that approximately 64%, 26% and 10% of ENU-induced single base pair mutations in coding regions are missense, synonymous, and nonsense mutations, respectively [28, 37, 38]. Intriguingly, the frequencies of base substitutions in the G1 exome decreased in the rank order of A/T to G/C transition > AT/TA transversion > G/C to A/T transition > G/C to T/A transversion > A/T to C/G transversion, consistent with estimates for the gene-driven approach [39]. Furthermore, the distribution of single base pair mutations in the G1 exome predicted to generate amino acid variants was approximately consistent with previous studies [37, 38].

Locomotor activity in the open field did not differ between L100P mutant mice (homozygotes and heterozygotes) and wild-type littermates, consistent with the report by Shoji et al. [27]. In contrast, Clapcote et al. [24] and Lipina et al. [25] reported higher locomotor activity in L100P/L100P mice than in wild-type littermates during the first 30 min, after introduction into the open field (but not thereafter). The higher locomotor activity of L100P mice may represent slower habituation to a novel environment. In addition, although Lipina et al. [25] reported higher locomotor activity in L100P mice following injection of amphetamine compared with that of wild-type littermates, we did not observe enhanced locomotion after an acute challenge with methamphetamine. Although the reasons for this discrepancy with previous reports remains to be explored, one possibility is that critical mutation(s) other than L100P may have been missed during maintenance of the strain after submission to the RIKEN EAD and/or the single backcrossing of homozygous (L100P/L100P) mutant males obtained from RIKEN to female inbred C57BL/6 J mice. As shown by the distribution of SNPs, the genetic background in the deposited L100P homozygotes was heterozygous; therefore, such heterogeneity may be fixed to either allele in the offspring, even after many generations of maintenance.

The enhanced release of amphetamine-evoked dopamine consistently reported in neuroimaging studies of schizophrenia [40–42] may be of relevance to this model of schizophrenia. However, Lipina et al. [25] did not find greater striatal release of dopamine following amphetamine injection in L100P mice. In the adult brain, *Disc1* is expressed in the hippocampus and not in the nucleus accumbens [43, 44]. The hyperactivity of subcortical dopaminergic neurons is believed to be conveyed by glutamatergic afferents from the hippocampus to the nucleus accumbens, which in turn regulates release of dopamine and the activity of dopaminergic neurons in the ventral tegmental area [45, 46]. Another possible mechanism relates to disruption of the interaction between the hippocampus and prefrontal cortex [47]. Although subtle cytoarchitectonic changes have been reported in frontal cortical neurons [48], *Disc1* L100P mice lack the reduced number of parvalbumin-positive interneurons observed in *Disc1* transgenic models [12, 18].

Sociability and social novelty preference tests used in the present study are used frequently to investigate disrupted social interactions in mouse models of schizophrenia [34]. The impoverished social interaction in *Disc1* mice is believed to be a relevant quantitative trait of social withdrawal in schizophrenia [14]. Consistent with Clapcote et al. [24], we found that the three L100P genotypes did not differ in sociability or social novelty preference. Intriguingly, sociability was markedly impaired in conditional transgenic lines with either constitutive expression of [14] or inducible expression of a mutant DISC1 C-terminal fragment during the early postnatal period [15]. We hypothesize that transgenic mice overexpressing human truncated DISC1 protein exhibit a dominant-negative effect that may lead to diminished binding between the DISC1 interacting proteins and relevant domains of DISC1 protein, thus altering the behavioral phenotype [15]. Based on these results, the relevance of *Disc1* L100P as a mouse model of schizophrenia should be re-evaluated.

A recent review stressed that ENU-mutagenized mice are useful for establishing novel models of complex human diseases, including neuropsychiatric diseases [49]. A range of ENU-generated mutations based on a phenotype-driven approach has an advantage over a complete loss-of-function mutation in recapturing the pleiotropic nature of human neuropsychiatric diseases and the subtlety of manifestation [49]. Thus, both disease-causative and other unexpected variants should be made publicly available [28, 29]. This phenotype-driven approach requires high-throughput behavioral screening to link mutation with function. One promising candidate screening methodology is the identification of outliers by neuroimaging. For example, only a few studies on developmental mouse models of psychiatric illness, including those focusing on DN-DISC1 models, have highlighted enlargement of the lateral ventricle without a marked change in overall brain size [16–18, 50]. Phenotype screening based on standardized behavioral tests [31, 51] combined with neuroimaging [50] to detect enlargement of the lateral ventricle may be an efficacious approach for the identification of robust schizophrenia models.

Several limitations of this study should be considered. First, we did not conduct a mouse comprehensive battery of behavioral tests including cognitive tasks and PPI but only tests of locomotor activity in a novel environment, psychostimulant-induced behavior and sociability in the three-chamber test. Therefore, our results do not clarify effects of the L100P amino acid substitution in exon 2 on other behavioral phenotypes related to psychiatric disorders including schizophrenia. Second, handling, experimental protocols, rearing environment and other environmental factors may influence behavioral test results and contribute to variability across studies [51, 52]. Third, L100P mice used in the current study were not genotyped for all ENU-induced mutations found in the exome and vicinity of the G1 genome, some of which may have been transmitted to progeny despite numerous backcrosses [24]. Given that 117 ENU-induced mutations in the G1 mouse identified with the whole-exome resequencing, it is plausible that 50-fold excess or more than 5,000 of ENU-induced mutations should exist in the entire G1 genome. Although detailed genetic information on models relevant to human disease should be freely available, routine implementation of whole-genome sequencing awaits measures for improved cost-effectiveness. However, as most of the ENU-induced mutations in G1 map to chromosomes other than chromosome 8 (containing *Disc1*), it is likely that they are transmitted to progeny to some extent regardless of the breeding process used to select mice harboring *Disc1* 100P. Although it may not be possible to investigate whether genetic background or other ENU-induced mutations have any effects on behavior because of a generalised lack of information on genotype differences among any of their mouse lines or offspring, further behavioral and genetic studies are warranted. For example, generation and breeding of another mouse line harboring *Disc1* L100P, which can be accomplished by *in vitro* fertilization by the cryopreserved sperm of the G1 progeny owned by RIKEN BRC, may facilitate full elucidation of a relationship between genetic components, including *Disc1* L100P mutation *per se*, and behavioral phenotypes, related with schizophrenia in this mouse line.

## Conclusion

The present study using behavioral genetic approaches provides an insight into the role of *Disc1* L100P and other single nucleotide variants in behavioral phenotypes associated with psychiatric disorders such as schizophrenia. Our present findings suggest that causal attribution of the discrepancy in behavioral phenotypes of the *Disc1* L100P mutant mouse line existing among different research groups, including our own, needs to be cautiously investigated in further studies by taking into account the effect(s) of other ENU-induced mutations and/or SNPs from DBA/2 J. Further behavioral genetic analyses are needed to elucidate the cause of behavioral variance associated with *Disc1* L100P strain. Using a polygenic animal model including ENU mutagenesis provides an efficacious approach to explore the relationship of variants to behavioral phenotypes associated with polygenic and multifactorial disorders such as psychiatric disorders.

## Electronic supplementary material

Additional file 1: Figure S1: Pedigree of Disc1 < Rgsc1390 > on RIKEN BRC. (PDF 29 KB)

Additional file 2: Table S1: Characteristics of mouse strain SNPs in Disc1<Rgsc1390>. (PDF 116 KB)
