# Supplementary figures and images for: Effects of background mutations and single nucleotide polymorphisms (SNPs) on the Disc1 L100P behavioral phenotype associated with schizophrenia in mice
Source: Behav Brain Funct. 2014 Dec 8;10:45. doi: 10.1186/1744-9081-10-45 (PMC4295473; doi:10.1186/1744-9081-10-45)

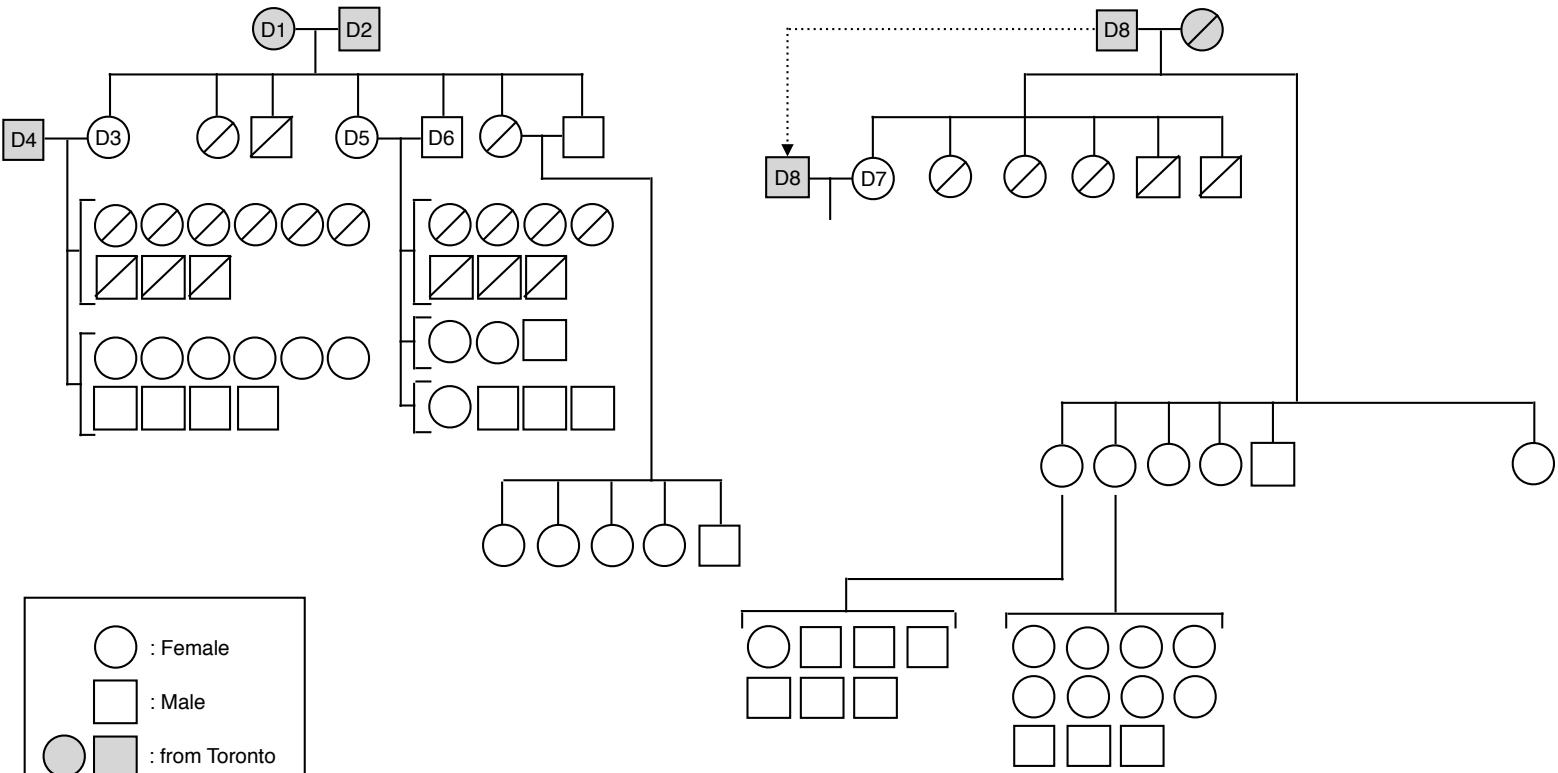

Supplement: Supplementary file 1 — Additional file 1: Figure S1: Pedigree of Disc1 < Rgsc1390 > on RIKEN BRC. (PDF 29 KB) [file 12993_2014_516_MOESM1_ESM.pdf]
