# Supplementary material for: Effects of background mutations and single nucleotide polymorphisms (SNPs) on the Disc1 L100P behavioral phenotype associated with schizophrenia in mice
Source: Behav Brain Funct. 2014 Dec 8;10:45. doi: 10.1186/1744-9081-10-45 (PMC4295473; doi:10.1186/1744-9081-10-45)
Supplement: Supplementary file 2 — Additional file 2: Table S1: Characteristics of mouse strain SNPs in Disc1. (PDF 116 KB) [file 12993_2014_516_MOESM2_ESM.pdf]

Table S1

Characteristics of mouse strain SNPs in Disc1<Rgsc1390>

			**Strain**	**C57BL/6JJcl**		**DBA/2J**		**D x B**		**Disc1<Rgsc1390>**								**Blank**	
			**No.**	1	2	3	4	5	6	D1	D2	D3	D4	D5	D6	D7	D8	7	8
**Chr**	**Position (MM9)**	**SNP ID**	**Marker name**																
1	4137358	rs3688428	D1SNP11	B6	B6	D2	D2	Het	Het	B6	B6	B6	B6	B6	B6	B6	B6	ND	ND
1	33302558	rs3690403	D1SNP12	B6	B6	D2	D2	Het	Het	B6	B6	B6	B6	B6	B6	B6	B6	ND	ND
1	67923485	rs3022803	D1SNP3	B6	B6	D2	D2	Het	Het	B6	B6	B6	B6	B6	B6	B6	B6	ND	ND
1	89100712	rs3022825	D1SNP3-1	B6	B6	D2	D2	Het	Het	B6	B6	B6	B6	B6	B6	B6	B6	ND	ND
1	123667033	rs3665018	D1SNP13	B6	B6	D2	D2	Het	Het	B6	B6	B6	B6	B6	B6	B6	B6	ND	ND
1	137337495	rs3022839	D1SNP5	B6	B6	D2	D2	Het	Het	B6	B6	B6	B6	B6	B6	B6	B6	ND	ND
1	173543848	rs3089480	D1SNP7	B6	B6	D2	D2	Het	Het	B6	B6	B6	B6	B6	B6	B6	B6	ND	ND
1	197134686	rs29609526	D1SNP27	B6	B6	D2	D2	Het	Het	B6	B6	B6	B6	B6	B6	B6	B6	ND	ND
2	7991482	rs13476337	D2SNP11	B6	B6	D2	D2	Het	Het	**Het**	**D2**	**Het**	**Het**	**Het**	**Het**	**D2**	**Het**	ND	ND
2	35967296	rs3703919	D2SNP12	B6	B6	D2	D2	Het	Het	B6	B6	B6	B6	B6	B6	B6	B6	ND	ND
2	54417652	rs3022884	D2SNP1	B6	B6	D2	D2	Het	Het	B6	B6	B6	B6	B6	B6	B6	B6	ND	ND
2	84442297	rs3022888	D2SNP3	B6	B6	D2	D2	Het	Het	B6	B6	B6	B6	B6	B6	B6	B6	ND	ND
2	106548159	rs27431854	D2SNP30	B6	B6	D2	D2	Het	Het	B6	B6	B6	B6	B6	B6	B6	B6	ND	ND
2	128915712	rs3022901	D2SNP4	B6	B6	D2	D2	Het	Het	B6	B6	B6	B6	B6	B6	B6	B6	ND	ND
2	159540910	rs3022937	D2SNP5	B6	B6	D2	D2	Het	Het	B6	B6	B6	B6	B6	B6	B6	B6	ND	ND
2	181454978	rs3022946	D2SNP6	B6	B6	D2	D2	Het	Het	B6	B6	B6	B6	B6	B6	B6	B6	ND	ND
3	5370727	rs13476956	D3SNP35	B6	B6	D2	D2	Het	Het	**Het**	B6	**Het**	B6	B6	**Het**	**Het**	B6	ND	ND
3	12512835	rs3661443	D3SNP11	B6	B6	D2	D2	Het	Het	B6	B6	B6	B6	B6	B6	B6	B6	ND	ND
3	37219744	rs3141187	D3SNP38	B6	B6	D2	D2	Het	Het	B6	B6	B6	B6	B6	B6	B6	B6	ND	ND
3	70996449	rs3704735	D3SNP13	B6	B6	D2	D2	Het	Het	B6	B6	B6	**Het**	B6	B6	**Het**	**Het**	ND	ND
3	100514332	rs3158070	D3SNP36	B6	B6	D2	D2	Het	Het	B6	B6	B6	**Het**	B6	B6	**Het**	**Het**	ND	ND
3	130054966	rs37019434	D3SNP37	B6	B6	D2	D2	Het	Het	B6	B6	B6	B6	B6	B6	B6	B6	ND	ND
3	148232054	rs3701592	D3SNP16	B6	B6	D2	D2	Het	Het	B6	B6	B6	B6	B6	B6	B6	B6	ND	ND
3	159387882	rs30101122	D3SNP34	B6	B6	D2	D2	Het	Het	B6	B6	B6	B6	B6	B6	B6	B6	ND	ND
4	3510322	rs27697629	D4SNP11	B6	B6	D2	D2	Het	Het	B6	B6	B6	B6	B6	B6	B6	B6	ND	ND
4	36411566	rs27818568	D4SNP109	B6	B6	D2	D2	Het	Het	B6	B6	B6	B6	B6	B6	B6	B6	ND	ND
4	68938181	rs13477759	D4SNP103	B6	B6	D2	D2	Het	Het	B6	B6	B6	B6	B6	B6	B6	B6	ND	ND
4	90787956	rs13477838	D4SNP104	B6	B6	D2	D2	Het	Het	B6	B6	B6	B6	B6	B6	B6	B6	ND	ND
4	125713065	rs13477964	D4SNP106	B6	B6	D2	D2	Het	Het	B6	B6	B6	B6	B6	B6	B6	B6	ND	ND
4	141429653	rs3716675	D4SNP16	B6	B6	D2	D2	Het	Het	B6	B6	B6	B6	B6	B6	B6	B6	ND	ND
4	155557887	rs6279100	D4SNP34	B6	B6	D2	D2	Het	Het	B6	B6	B6	B6	B6	B6	B6	B6	ND	ND
5	7163511	rs3090822	D5SNP1-1	B6	B6	D2	D2	Het	Het	B6	B6	B6	B6	B6	B6	B6	B6	ND	ND
5	24619610	rs33648900	D5SNP11	B6	B6	D2	D2	Het	Het	B6	B6	B6	B6	B6	B6	B6	B6	ND	ND
5	52907865	rs3090699	D5SNP1-3	B6	B6	D2	D2	Het	Het	B6	B6	B6	B6	B6	B6	B6	B6	ND	ND
5	83810839	rs13478361	D5SNP12	B6	B6	D2	D2	Het	Het	B6	B6	B6	B6	B6	B6	B6	B6	ND	ND
5	115597885	rs3719767	D5SNP13	B6	B6	D2	D2	Het	Het	B6	B6	B6	B6	B6	B6	B6	B6	ND	ND
5	150428596	rs33208334	D5SNP14	B6	B6	D2	D2	Het	Het	**Het**	**Het**	**Het**	**Het**	**Het**	B6	**Het**	**Het**	ND	ND
6	3695835	rs30662637	D6SNP19	B6	B6	D2	D2	Het	Het	B6	B6	B6	B6	B6	B6	B6	B6	ND	ND
6	17166128	rs3664283	D6SNP11	B6	B6	D2	D2	Het	Het	B6	B6	B6	B6	B6	B6	B6	B6	ND	ND
6	28665963	rs30764547	D6SNP20	B6	B6	D2	D2	Het	Het	**Het**	**Het**	**Het**	**Het**	**D2**	**D2**	**D2**	**D2**	ND	ND
6	55398977	rs3023071	D6SNP2	B6	B6	D2	D2	Het	Het	B6	B6	B6	B6	B6	B6	B6	B6	ND	ND
6	88800146	rs29973762	D6SNP24	B6	B6	D2	D2	Het	Het	B6	B6	B6	B6	B6	B6	B6	B6	ND	ND
6	122265814	rs3023094	D6SNP5	B6	B6	D2	D2	Het	Het	B6	B6	B6	B6	B6	B6	B6	B6	ND	ND
6	146425319	rs3023105	D6SNP6	B6	B6	D2	D2	Het	Het	B6	B6	B6	B6	B6	B6	B6	B6	ND	ND
7	4318466	rs3714976	D7SNP11	B6	B6	D2	D2	Het	Het	B6	B6	B6	B6	B6	B6	B6	B6	ND	ND
7	30383795	rs3671361	D7SNP15	B6	B6	D2	D2	Het	Het	B6	B6	B6	B6	B6	B6	B6	B6	ND	ND
7	62107793	rs31830738	D7SNP30	B6	B6	D2	D2	Het	Het	B6	B6	B6	B6	B6	B6	B6	B6	ND	ND
7	98302717	rs3023148	D7SNP4	B6	B6	D2	D2	Het	Het	ND	B6	B6	B6	B6	B6	B6	B6	ND	ND
7	136179208	rs13479522	D7SNP37	B6	B6	D2	D2	Het	Het	**Het**	**Het**	B6	**D2**	**Het**	B6	**Het**	**Het**	ND	ND
7	152073221	rs3692548	D7SNP14	B6	B6	D2	D2	Het	Het	B6	B6	B6	B6	B6	B6	B6	B6	ND	ND
8	9530463	rs13479603	D8SNP13	B6	B6	D2	D2	Het	Het	B6	B6	B6	B6	B6	B6	B6	B6	ND	ND
8	35491057	rs3023185	D8SNP2	B6	B6	D2	D2	Het	Het	B6	B6	B6	B6	B6	B6	B6	B6	ND	ND
8	73898167	rs3023188	D8SNP4	B6	B6	D2	D2	Het	Het	B6	B6	B6	B6	B6	B6	B6	B6	ND	ND
8	99037614	rs32192192	D8SNP11	B6	B6	D2	D2	Het	Het	B6	B6	B6	B6	B6	B6	B6	B6	ND	ND
8	125851611	rs33405503	D8SNP12	B6	B6	D2	D2	Het	Het	B6	B6	B6	B6	B6	B6	B6	B6	ND	ND
9	6215905	rs29982480	D9SNP11	B6	B6	D2	D2	Het	Het	**Het**	**Het**	B6	**Het**	**Het**	**Het**	**Het**	**Het**	ND	ND
9	33606003	rs3023206	D9SNP1-1	B6	B6	D2	D2	Het	Het	B6	B6	B6	B6	B6	B6	B6	B6	ND	ND
9	48612132	rs3023210	D9SNP3	B6	B6	D2	D2	Het	Het	B6	B6	B6	B6	B6	B6	B6	B6	ND	ND

9	73461716	rs13480275	D9SNP20	B6	B6	D2	D2	Het	Het	B6	B6	B6	B6	B6	B6	B6	B6	ND	ND
9	98346928	rs3091100	D9SNP4	B6	B6	D2	D2	Het	Het	B6	B6	B6	B6	B6	B6	B6	B6	ND	ND
9	121500286	rs3660182	D9SNP12	B6	B6	D2	D2	Het	Het	B6	B6	B6	B6	B6	B6	B6	B6	ND	ND
10	5540158	rs29362926	D10SNP11	B6	B6	D2	D2	Het	Het	B6	B6	B6	B6	B6	B6	B6	B6	ND	ND
10	28315853	rs3023233	D10SNP2	B6	B6	D2	D2	Het	Het	B6	**D2**	**Het**	**D2**	**Het**	**Het**	B6	**Het**	ND	ND
10	33372829	rs13480575	D10SNP36	B6	B6	D2	D2	Het	Het	B6	**Het**	B6	**Het**	B6	B6	B6	**Het**	ND	ND
10	67604188	rs3680724	D10SNP12	B6	B6	D2	D2	Het	Het	B6	B6	B6	B6	B6	B6	B6	B6	ND	ND
10	107291729	rs3655255	D10SNP13	B6	B6	D2	D2	Het	Het	B6	**D2**	**Het**	**Het**	**Het**	**Het**	**D2**	**Het**	ND	ND
10	127251537	rs29363885	D10SNP34	B6	B6	D2	D2	Het	Het	B6	B6	B6	B6	B6	B6	B6	B6	ND	ND
11	4408733	rs3659787	D11SNP101	B6	B6	D2	D2	Het	Het	**D2**	B6	**Het**	B6	**Het**	**Het**	**Het**	B6	ND	ND
11	44269852	rs3023256	D11SNP3	B6	B6	D2	D2	Het	Het	B6	B6	B6	**Het**	B6	B6	B6	B6	ND	ND
11	75418964	rs3714172	D11SNP11	B6	B6	D2	D2	Het	Het	B6	B6	B6	B6	B6	B6	B6	B6	ND	ND
11	99370140	rs3023315	D11SNP7	B6	B6	D2	D2	Het	Het	B6	B6	B6	B6	B6	B6	B6	B6	ND	ND
11	117028591	rs3023316	D11SNP8	B6	B6	D2	D2	ND	Het	B6	B6	B6	B6	B6	B6	B6	B6	ND	ND
12	5593905	rs29122374	D12SNP11	B6	B6	D2	D2	Het	Het	B6	B6	B6	B6	B6	B6	B6	B6	ND	ND
12	30739697	rs3023342	D12SNP2	B6	B6	D2	D2	ND	Het	B6	B6	B6	B6	B6	B6	B6	B6	ND	ND
12	59523071	rs31975474	D12SNP34	B6	B6	D2	D2	Het	Het	B6	B6	B6	B6	B6	B6	B6	B6	ND	ND
12	88536832	rs29490233	D12SNP35	B6	B6	D2	D2	Het	Het	B6	B6	B6	B6	B6	B6	B6	B6	ND	ND
12	117628255	rs3718956	D12SNP14	B6	B6	D2	D2	Het	Het	B6	B6	B6	B6	B6	B6	B6	B6	ND	ND
13	6303301	rs13481676	D13SNP11	B6	B6	D2	D2	Het	Het	**Het**	**D2**	**Het**	**D2**	**Het**	**D2**	**D2**	**D2**	ND	ND
13	33139225	rs3023380	D13SNP2	B6	B6	D2	D2	Het	Het	B6	B6	B6	B6	B6	B6	B6	B6	ND	ND
13	67061452	rs6283060	D13SNP25	B6	B6	D2	D2	Het	Het	B6	B6	B6	B6	B6	B6	B6	B6	ND	ND
13	103496680	rs3090371	D13SNP4	B6	B6	D2	D2	Het	Het	B6	B6	B6	B6	B6	B6	B6	B6	ND	ND
13	120251113	rs3704669	D13SNP24	B6	B6	D2	D2	Het	Het	B6	B6	B6	B6	B6	B6	B6	B6	ND	ND
14	8784057	rs4230140	D14SNP22	B6	B6	D2	D2	Het	Het	B6	B6	B6	B6	B6	B6	B6	B6	ND	ND
14	14457541	rs30577733	D14SNP11	B6	B6	D2	D2	Het	Het	B6	B6	B6	B6	B6	B6	B6	B6	ND	ND
14	46359100	rs3683618	D14SNP12	B6	B6	D2	D2	Het	Het	**Het**	**Het**	B6	**Het**	**Het**	**Het**	B6	**Het**	ND	ND
14	71665765	rs3724044	D14SNP13	B6	B6	D2	D2	Het	Het	**Het**	**Het**	B6	**Het**	**Het**	**Het**	**Het**	**Het**	ND	ND
14	106810168	rs3023414	D14SNP6	B6	B6	D2	D2	Het	Het	B6	B6	B6	B6	B6	B6	B6	B6	ND	ND
14	121511809	rs30791519	D14SNP21	B6	B6	D2	D2	Het	Het	B6	B6	B6	B6	B6	B6	B6	B6	ND	ND
15	4252715	rs31764373	D15SNP26	B6	B6	D2	D2	Het	Het	B6	B6	B6	B6	B6	B6	B6	B6	ND	ND
15	32625942	rs3091174	D15SNP1	B6	B6	D2	D2	Het	Het	B6	B6	B6	B6	B6	B6	B6	B6	ND	ND
15	56992041	rs3702158	D15SNP24	B6	B6	D2	D2	Het	Het	**Het**	**Het**	**D2**	B6	**Het**	**Het**	**Het**	**Het**	ND	ND
15	79555919	rs13459189	D15SNP25	B6	B6	D2	D2	Het	Het	B6	B6	B6	B6	B6	B6	B6	B6	ND	ND
15	103136059	rs3698193	D15SNP14	B6	B6	D2	D2	Het	Het	B6	B6	B6	B6	B6	B6	B6	B6	ND	ND
16	5104751	rs4152847	D16SNP17	B6	B6	D2	D2	Het	Het	B6	B6	B6	B6	B6	B6	B6	B6	ND	ND
16	10741068	rs3090882	D16SNP2	B6	B6	D2	D2	Het	Het	B6	B6	B6	B6	B6	B6	B6	B6	ND	ND
16	38931238	rs3089786	D16SNP3	B6	B6	D2	D2	Het	Het	B6	B6	B6	B6	B6	B6	B6	B6	ND	ND
16	66149007	rs4197051	D16SNP18	B6	B6	D2	D2	Het	Het	B6	B6	B6	B6	B6	B6	B6	B6	ND	ND
16	93319624	rs3722671	D16SNP12	B6	B6	D2	D2	Het	Het	B6	B6	B6	B6	B6	B6	B6	B6	ND	ND
17	3223874	rs33085767	D17SNP23	B6	B6	D2	D2	Het	Het	B6	B6	B6	B6	B6	B6	B6	B6	ND	ND
17	15302144	rs33390998	D17SNP11	B6	B6	D2	D2	Het	Het	B6	B6	B6	B6	B6	B6	B6	B6	ND	ND
17	34546881	rs3023444	D17SNP3	B6	B6	D2	D2	Het	Het	B6	B6	B6	B6	B6	B6	B6	B6	ND	ND
17	66897325	rs3708432	D17SNP12	B6	B6	D2	D2	Het	Het	B6	B6	B6	B6	B6	B6	B6	B6	ND	ND
17	93740098	rs3674428	D17SNP13	B6	B6	D2	D2	Het	Het	B6	B6	B6	B6	B6	B6	B6	B6	ND	ND
18	6422727	rs29955209	D18SNP11	B6	B6	D2	D2	Het	Het	B6	B6	B6	B6	B6	B6	B6	B6	ND	ND
18	36955024	rs13489298	D18SNP32	B6	B6	D2	D2	Het	Het	B6	B6	B6	B6	B6	B6	B6	B6	ND	ND
18	57453544	rs13483378	D18SNP34	B6	B6	D2	D2	Het	Het	B6	B6	B6	B6	B6	B6	B6	B6	ND	ND
18	86273032	rs3702803	D18SNP13	B6	B6	D2	D2	Het	Het	B6	B6	B6	B6	B6	B6	B6	B6	ND	ND
19	4311697	rs3677505	D19SNP11	B6	B6	D2	D2	Het	Het	B6	B6	B6	B6	B6	B6	B6	B6	ND	ND
19	22628647	rs13483566	D19SNP13	B6	B6	D2	D2	Het	Het	B6	B6	B6	B6	B6	B6	B6	B6	ND	ND
19	47191291	rs3090751	D19SNP3	B6	B6	D2	D2	Het	Het	B6	B6	B6	B6	B6	B6	B6	B6	ND	ND
19	57583460	rs30326084	D19SNP12	B6	B6	D2	D2	Het	Het	B6	B6	B6	B6	B6	B6	B6	B6	ND	ND
20	8991149	rs3725913	DXSNP11	B6	B6	D2	D2	Het	Het	B6	B6	B6	**D2**	B6	B6	B6	B6	ND	ND
20	47850822	rs3679178	DXSNP12	B6	B6	D2	D2	Het	Het	**Het**	B6	**Het**	B6	B6	B6	B6	B6	ND	ND
20	73554577	rs3695066	DXSNP13	B6	B6	D2	D2	Het	Het	**Het**	**D2**	**D2**	B6	**Het**	B6	B6	B6	ND	ND
20	116938586	rs31262455	DXSNP16	B6	B6	D2	D2	Het	Het	B6	B6	B6	B6	B6	B6	B6	B6	ND	ND
20	134464194	rs29262348	DXSNP46	B6	B6	D2	D2	Het	Het	B6	B6	B6	B6	B6	B6	B6	B6	ND	ND
20	160951484	rs4136868	DXSNP17	B6	B6	D2	D2	Het	Het	B6	B6	B6	B6	B6	B6	B6	B6	ND	ND

D x B: DBA/2J x C57BL/6J, B6: homozygous SNP derived from C57BL/6JJcl strain, D2: homozygous SNP derived from DBA/2J strain, Het: heterozygote, ND: not detected
